# Facts and Factors in Medicine

**Published:** 1911-09-02

**Authors:** 


### FACTS AND FACTORS IN MEDICINE.

### SOME PRELIMINARY CONSIDERATIONS FOR THE STUDENT.

Exactly what the future of the medical profession is going to be during the next two generations is a difficult matter to forecast. Both professionally and commercially (and ethically as well), there are changes in the air the ultimate issue of which no man can entirely and accurately predict. Most members of the profession believe that the conditions under which medical practice has hitherto been carried on will prevail no longer, and the majority view with some apprehension the result of the recasting of the foundations of medicine as a profession. Apart altogether from the provisions of the Insurance Bill, whose effect will not be discussed here, it is also to be remembered that many other factors of recent growth have altered, and will continue to alter, the relations of the art of medicine to the general polity of the commonwealth. The growth of specialism; its popularity with the upper and middle classes; the expansion of the newly arisen branches of medicine, such as bacteriology, electro-therapeutics, radium-therapy, vaccine-therapy, and the rest; the development of modern surgery; the advances of hygiene and preventive medicine—all these have modified profoundly the position of the medical profession in relation to its clients and to the world at large. Moreover the same developments have provided work for a considerable and ever-increasing percentage of those who complete the medical curriculum and obtain admission to the Register. It is apparent, therefore, that the career of medicine may now (and in fact does) attract and provide work for many men who have no aptitude for general or for special practice; far more so than was the case a few years ago. The man with a squint, an incurable stutter, a lame leg, or a glass eye, need no longer be considered as debarred from the study of medicine if he desires to adopt it; for the last is no more of a hindrance to a medical officer of health than the first is to a bacteriologist, all other things being equal.

#### What Medicine Demands.

We may put aside, therefore, for the present the question whether or not it is advisable to commence medical study, with a view to becoming in due time a medical practitioner. We shall assume that the decision is already made, and that the question of the moment is how, not whether. How can the intending doctor achieve his ambition? In many ways. Which way is the best? That depends on many things; upon means, capacity, age, inclination, locality, and many other influences.

First of all, it must be clearly understood that to become a qualified medical man is a task which is beyond the power of practically no one, however stupid he may be, so long as he is *compos mentis*. A man who from sheer lack of intelligence, and from that cause alone, fails to obtain admission eventually to the Medical Register, is a man for whom failure is quite certain in every other walk of life. Yet only about 50 per cent. of those who commence medical study ever qualify—a weeding out of the unfit greater probably than in any other profession to which examination tests are applied. Notwithstanding this, we reassert with confidence that the most ordinary ability will suffice to obtain the recognition which is implied by qualification. It is much more a matter of application than of brains. It cannot of course be denied that some men gain their qualifying degree or diploma with much less effort than others. But it is equally true that work, and plenty of it, is the one essential royal road to qualification; and that no one who has really set his heart on becoming a doctor need fail while his health, his mind, and his funds hold out.

#### Work and Character.

Let no mistake be entertained upon this score. The growth of the curriculum, consequent upon the enormous expansion of scientific medicine and surgery in recent years, has been such that no idler can ever encompass it. It takes years of good, solid hard work to become a qualified practitioner, and it takes constant and even harder work afterwards to succeed in the profession, no matter what branch of it be adopted. Medicine is no career for an idler; everyone cannot be a genius, no doubt, but everyone can work, and those who can and will work are those alone for whom medicine holds out her rewards. The other essential asset of the future medical man, besides willingness to work, is character. These two traits rank far ahead of all others among the requisites for success in the medical profession. Having them, the doctor has the world at his feet for his football; he may be assured of moral and material success.

#### The Avenues to Qualification.

The parent or guardian of a lad for whom a medical career has been determined on has several anxious and important questions to settle right at the outset. Among these are the degree or diploma at which the student shall aim, and the school or schools at which he shall be trained for it. The answer to the latter question depends so much upon the answer to the former, that its consideration should be deferred until a decision has been arrived at concerning the degree or diploma to be attempted. This brings us at once to the trite but very important question of the relative values of the various qualifying examinations which entitle to registration. Opinions differ over this vexed question, but the views which have always been advocated in The Hospital have commended themselves to a majority of the profession, so they will be briefly restated and recapitulated.

There are certain medical qualifying examinations (or rather certain series of examinations) which require more preparation, more assiduity, more intellect; and there are others which require less. To venture on comparisons may offend local susceptibilities, but the proposition just stated may be exemplified without offence by instancing the Fellowship of the Royal College of Surgeons and the Licentiateship of the Society of Apothecaries. Yet there are undoubtedly some men whose sole title to registration is the L.S.A., to whose hands most of their medical acquaintances would sooner entrust their own lives than to some men who can write themselves F.R.C.S. These are exceptional cases, and in the main it cannot be disputed that the average Fellow of the Royal College of Surgeons is a more expert member of the profession than the average Licentiate of the Society of Apothecaries. And in point of fact the instructed public recognises this, though there are of course millions of the uninstructed who do not know one medical qualification from another. Further, in all public appointments, the one diploma carries far more weight than the other; in fact, commercially it is a far more valuable and a far more marketable asset.

### The Degree of M.D.

We have taken so far the extreme case, where the dividing line is easy to draw, though even then it does not constitute an infallible guide to the practical sagacity, the tact, or the experience of the owners of the two diplomas. When we come to the multifarious other degrees and diplomas the difficulty is multiplied a hundredfold. Thus the M.D. of London University has secured in lay esteem a place of vantage which is long established and unassailable. Without doubt the preliminary examinations for that degree are a severe test of ability and of application; and the final examinations in professional subjects are also kept up to a high standard. Still for all that it is quite certain that the significance of this degree is over-estimated by the general public, and that the bookish student who can encompass it with ease frequently fails to succeed in practice to the same extent as his fellows who have essayed it in vain. Whether this is the fault of the examination or of the examiners is not easy to say, though certainly it has been alleged that the examiners encourage abstruse and doctrinaire learning too much, at the expense of practical training and experience.

There are numerous other M.D. degrees, some more esteemed in the profession, some less. The doctorates of Oxford and Cambridge carry the prestige both of a high level of mere scholastic proficiency and of a university training as well. They are justly esteemed very highly on both grounds, and the latter especially is characterised by the importance which is attached to evidence of technical and practical familiarity with the common problems of medical and surgical practice. The Edinburgh M.D. is another which has an ancient reputation and maintains it worthily.

There are, however, several universities in Britain, some new, some not new, where the degree of M.D. is granted after exercises much less strenuous than at the seminaries just enumerated. The graduates of these universities are naturally somewhat jealous of any belittlement of their *almæ matres*, and resent any criticism of the standard of their degrees. Yet beyond doubt it is possible thus to become an M.D. with no more expenditure of time, trouble, and money than suffices elsewhere to procure a diploma, and a diploma only. The graduates of these universities thus obtain some of the advantages which arise from the public respect for an M.D. degree without themselves having done anything to maintain that public respect. They obtain, in a worldly sense, an undeserved advantage over their contemporaries from other schools. It is not, of course, that their training and their curriculum and their examinations are not good; the Medical Council see to that. But they are not of the same high standard of excellence of the other universities, and they are not of a higher standard than (if, indeed, always of as high as) those of some of the diploma-issuing corporations. The best known of the latter—indeed, much the largest body of registered practitioners in the kingdom—is the Conjoint Board of the Royal Colleges of Physicians and Surgeons in England. This Board conducts examinations which entitle those who pass them to subscribe themselves M.R.C.S., L.R.C.P., a title which, on account of not being a degree, is treated with unmerited contempt by one section of the community, and on account of its containing so many letters with exaggerated respect by another section. The plain fact of the matter is that these examinations are not so searching and so difficult to pass as are some of the better kind of degree examinations; but they are of much the same kind and standing as those of the less exigent universities. In all cases it must be fully understood that *as tests of sound and practical clinical wisdom* examinations of all sorts are very fallacious. There are scores, nay hundreds, of Conjoint Board diploma men whose ability as physicians and surgeons is altogether excellent, just as there are plenty of degree men whose talents and aptitude for their profession are but mediocre. Reversely, there are many men with diplomas whose intellects never could give them the hope of either passing a stiff M.D. examination or of making a deserved reputation for themselves in after life as practitioners. One fact is certain, however, that with the general public there is a certain glamour about an M.D. degree, however obtained, which makes its possession a profitable investment of time and money.

### The Choice of a Qualification.

To return, then, to the practical question confronting the future medical student and his advisers, What goal should be sought? With many the answer is predetermined by means, or the lack of them. The nearest or the cheapest medical school must perforce be selected, and the shortest curriculum must be taken which will enable the student to qualify and earn his living speedily. Speaking generally, this means for anyone living near London the Conjoint Board diploma obtained by medical study at one or other of the numerous medical schools in London; in the provinces it means study at one or other of the universities which are now dotted about the map of England, with either the Conjoint Board diploma or the local degree as the object in view. In Scotland it means resort to one or other of the universities with which she has always been plentifully endued, and either the Scottish diplomas or the local university degree as the qualifying portal to the Medical Register; and similarly with Wales and Ireland. For those thus situated must make a virtue of necessity, and freedom of choice is to some extent necessarily denied them.

Let us consider, however, the case of a man to whom fortune has been rather more kind than this, a man who does not need to bustle himself on to the Register in the absolute minimum of time and at the lowest possible expenditure. On much the same footing, too, stands he who, though lacking means, has it in his power to earn a little money during his studentship, be it by a talent for music, for journalism, or by any other gift which can be translated into currency; and along with these fortunate individuals we may also consider those whose industry or ability enables them to smooth their own paths financially by means of prizes, exhibitions, and scholarships. Such as these need carefully to consider, first, how far their means will carry them; secondly, how long they can devote to preparation before they start to earn money; and thirdly, what precisely they propose to aim at.

Those who are lucky enough to afford a university career—by which, for the moment, is meant one at Oxford or Cambridge—are well advised not to let slip such an opportunity. True, it means deferring by at least a year (and probably by two years) the ultimate date of qualification; but the advantages of a university education are still so great that from a purely mercenary standpoint alone they are well worth having. But before such a programme is entered upon it should be clearly understood that the cost will not be light—though, of course, there are many opportunities of diminishing it by means of scholarships. To take an arts course at Oxford or Cambridge and to carry on medical study at the same time, which is the usual plan of medical students, and then to finish the necessary clinical work at some other medical school (usually in London) will take even the most capable and energetic young men about six years and cost not less than £1,000; most probably the cost will be even more than this, and if a longer time be taken over the curriculum, the expenses will be proportionately increased. It is further to be borne in mind that the regulation course of medical study, terminating in a successful ordeal by examination, is far from equipping the young doctor completely for practice. Whenever such a thing is at all possible, it is of the utmost benefit that the newly qualified practitioner should devote a year or two to the holding of resident hospital appointments. The best of these—at any rate, those most sought after—are those at the hospitals to which teaching schools are attached; and these are as a rule honorary. So that for a further period after qualification the young doctor who takes advantage of the unique and valuable experience thus to be gained is still at his own charges. If a resident appointment be taken elsewhere than at a teaching hospital, some small salary is usually attached, ranging from £40 to £120 a year; in no case is the remuneration at all commensurate with the advantages which the hospital derives from the resident's services. It will thus be realised that an Oxford or Cambridge degree is not attainable by everyone.

Those who, while not able to afford such an extended and expensive training as this, are yet in a position to please themselves to some extent, must select a medical school. In this propinquity, reputation, association, and personal recommendation are all of moment. To recommend one medical school or another is no part of the present purpose, but when the school has been chosen certain general principles should be followed in arranging the course of study. To begin with, it is always advisable to start with a high ambition; nothing is more disappointing than to achieve an easy qualification and then to find the road to further success barred by the omission to pass the preliminary stages for the higher examinations. It is very tempting to pass on at once to clinical study after completing the examinations in anatomy and physiology for some degree or diploma without grinding away for some months more in order to pass the "primary Fellowship"; but it is a fatal mistake to do this, if there is the remotest possibility of adopting surgery as a specialty later on, for then is entailed the irksome labour of going back to the laboratory and the dissecting-room to get up the elaborate minutiæ of half-forgotten sciences in order to go for the final Fellowship itself.

If medical study is being pursued in London, it is well to proceed to the examinations of the London M.B. as they fall due, instead of being content merely with those of the "colleges." If in the provinces the student should adopt either the degree course of his local university or that of the University of London, he should remember that in practically all the more difficult degrees and qualifications the preliminary and intermediate examinations are more difficult of their kind than the finals. Anyone who can pass the Intermediate M.B. London, or the primary Fellowship, for example, is certain to pass the respective finals if he sticks to work with the same intensity.

Lastly, it should be well impressed on students that examinations and degrees are only means to an end, not an end in themselves. The end in view is the perfecting of oneself in one's art to the utmost possible degree within one's own limitations. Degrees and qualifications are useful in so far as they are necessary preliminaries to practice, and to appointments of various kinds which are opportunities for practice. But it is honest work, and an appreciation of the fact that the medical man is a student till the day he dies, which really make the conscientious doctor an asset to the State. If all those who wish to enter the profession will realise this and determine that this ideal is what they are making up their minds to live up to, there need be little fear that the next generation of medical men will fail to carry on those honourable traditions which the present-day members have received from their predecessors and which they intend to hand on undimmed and unstained to their successors.

